# Assessment of temporospatial and kinematic gait parameters using human pose estimation in patients with Parkinson’s disease: A comparison between near-frontal and lateral views

**DOI:** 10.1371/journal.pone.0317933

**Published:** 2025-01-24

**Authors:** Jeongsik Kim, Ryul Kim, Kyeongho Byun, Nyeonju Kang, Kiwon Park

**Affiliations:** 1 Department of Biomedical and Robotics Engineering, Incheon National University, Incheon, Korea; 2 Department of Neurology, Seoul Metropolitan Government ‐ Seoul National University Boramae Medical Center, Seoul National University College of Medicine, Seoul, Korea; 3 Division of Sport Science, Sport Science Institute & Health Promotion Center, Incheon National University, Incheon, Korea; University of Tehran, ISLAMIC REPUBLIC OF IRAN

## Abstract

Gait disturbance is one of the most common symptoms in patients with Parkinson’s disease (PD) that is closely associated with poor clinical outcomes. Recently, video-based human pose estimation (HPE) technology has attracted attention as a cheaper and simpler method for performing gait analysis than marker-based 3D motion capture systems. However, it remains unclear whether video-based HPE is a feasible method for measuring temporospatial and kinematic gait parameters in patients with PD and how this function varies with camera position. In this study, treadmill and overground walking in 24 patients with early PD was measured using a motion capture system and two smartphone cameras placed on the near-frontal and lateral sides of the subjects. We compared the differences in temporospatial gait parameters and kinematic characteristics between joint position data obtained from the 3D motion capture system and the markerless HPE. Our results confirm the feasibility of analyzing gait in patients with PD using HPE. Although the near-frontal view, where the heel and toe are clearly visible, is effective for estimating temporal gait parameters, the lateral view is particularly well-suited for assessing spatial gait parameters and joint angles. However, in clinical settings where lateral recordings are not feasible, near-frontal view recordings can still serve as a practical alternative to motion capture systems.

## Introduction

Gait impairment is a cardinal feature of Parkinson’s disease (PD) and can manifest in the early and even prodromal stages of the disease [1, 2]. PD patients commonly exhibit reduced gait speed, shortened stride length, and increased double support time compared to healthy individuals [3]. Because the presence of gait impairment is associated with functional independence, poor health-related quality of life, and increased risk of falls and death in patients with PD [4], it is crucial to closely identify and monitor this condition. Currently, clinician-dependent scales or questionnaires for gait, such as the Movement Disorders Society-Unified PD Rating Scale (MDS-UPDRS) part III, Freezing of Gait Questionnaire, and Tinetti Gait and Balance instrument, are widely used in both clinical and research settings [5]. However, these tests are limited by their semiquantitative nature and test. Although there have been many attempts to evaluate gait parameters objectively using wearable sensors or pressure sensor mats, such methods have accessibility limitations because of device requirements and setup expertise [6].

The widely accepted standard for gait analysis is 3D motion capture, in which markers are attached to the body to record movement. However, motion capture has limitations, including high equipment costs, the need for specialized personnel to take measurements, and the need for a large measurement space. In contrast, computer vision based 2D human pose estimation (HPE) overcomes these limitations to provide a more practical and accessible alternative. In particular, MediaPipe Pose is a lightweight model compared to the more calculation cost-demanding OpenPose and has the advantage of real-time data processing on both mobile and desktop platforms. It also requires no camera synchronization or calibration, making it easy to apply in a variety of environments [7].

Computer vision technology is advancing rapidly in neurology, particularly in motor function assessment and disease monitoring. HPE holds potential for gait analysis and motor assessments in neurological disorders, including PD. However, to enable broader clinical adoption, these techniques must be validated for accuracy and reliability against traditional motion capture systems. Previous studies have demonstrated clinical gait analysis using video-based posture estimation for different perspectives, clinical populations, and measures of change, highlighting the growing potential of 2D video-based tracking for gait spatiotemporal parameters and kinematic analysis in PD [8]. OpenPose and Mediapipe Pose are widely used as marker-free technologies for gait analysis. Mediapipe is a lighter model and has strengths in real-time processing, while OpenPose is characterized by providing high accuracy [9, 10]. Computer vision has been effectively used to automate gait analysis, as demonstrated by a pilot study that successfully extracted spatiotemporal parameters and identified gait abnormalities without physical markers [11]. Another study employed machine learning algorithms to classify gait patterns and predict fall risks, further highlighting the potential of combining computer vision with AI to enhance clinical gait analysis [12].

Tracking of 2D videos with pose-estimation methods has emerged as a viable approach for objectively measuring gait impairment in PD [13–15]. Recently, this approach has been validated for measuring gait patterns in patients with PD [16]. In this study, we evaluated the accuracy of 2D gait video-based analysis using pose estimation to measure temporospatial gait parameters and joint kinematics from near-frontal and lateral views obtained from MediaPipe Pose, comparing the results with those obtained from a 3D motion capture system in patients with PD. To ensure the results reflect realistic clinical conditions, this study was intentionally designed to omit camera calibration, emphasizing the practicality and applicability of markerless HPE in real-world clinical settings.

## Methods

### Study design and participants

In this study, we used baseline gait data of 24 patients with PD obtained from an exercise intervention pilot trial that was approved by the Institutional Review Board of the Inha University Hospital (2022-01-030) and registered at cris.nih.go.kr (KCT0007130) [17, 18]. All methods were performed in accordance with the Declaration of Helsinki. Written informed consent was obtained from all the enrolled patients. The inclusion criteria were patients with PD who had Hoehn and Yahr stage ≤2 and a disease duration <5 years. PD was diagnosed based on the UK PD Society Brain Bank Diagnostic Criteria. Patients with neurological, orthopedic, or cardiovascular conditions deemed unsuitable for aerobic exercise were excluded from participation in the study. The gait data contained 2D video recordings of near-frontal and lateral views captured on a smartphone as well as 3D motion capture system data.

### Experimental design

**Fig 1:**
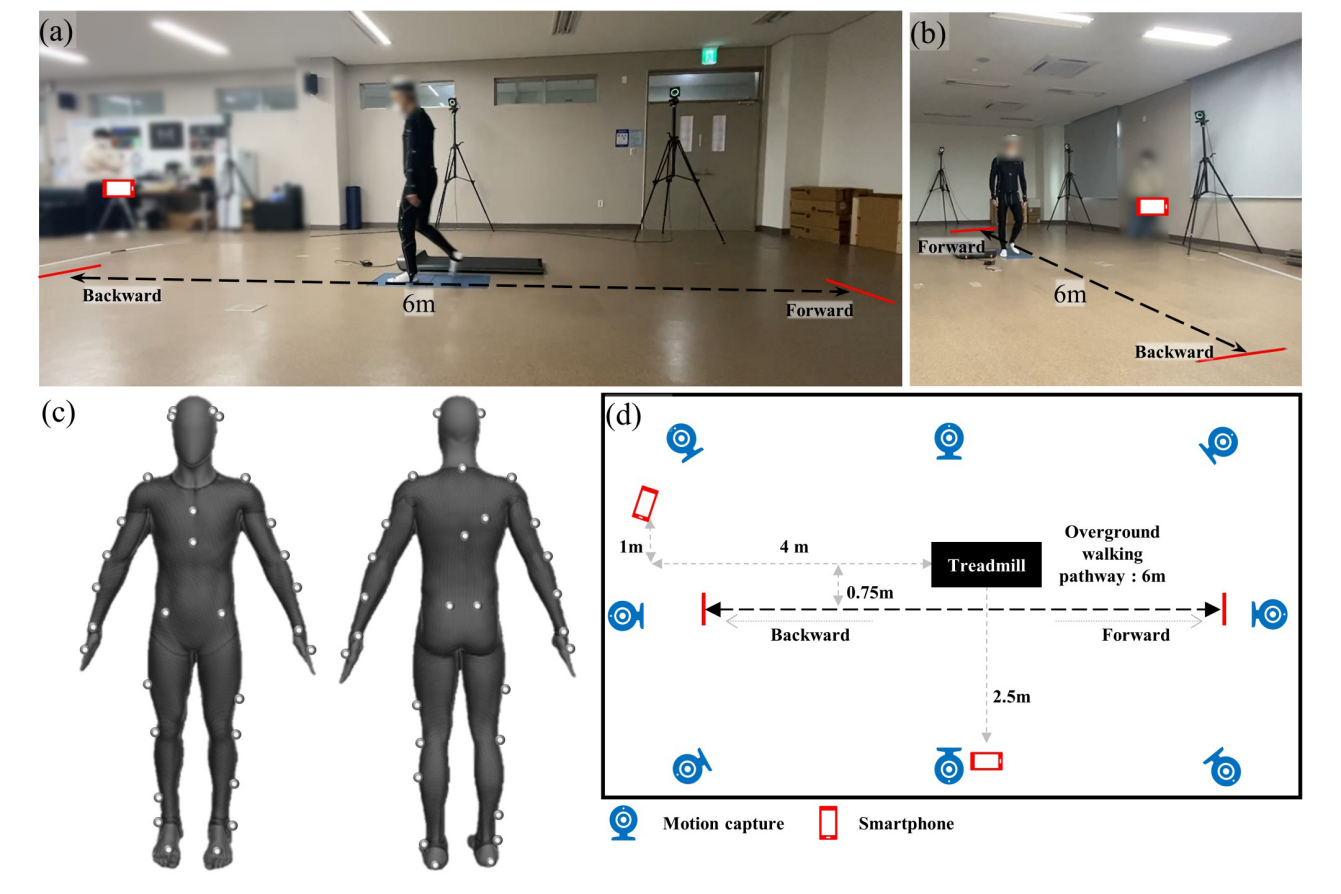
(a) Lateral view of the subject’s gait during the experiment. (b) Near-frontal view of the subject’s gait during the experiment. (c) Experimental setup: The arrangement of the treadmill and cameras for recording gait, with arrows indicating the walking direction. (d) Whole-body marker set used for motion capture, as provided by Motive software.

Gait data of 24 patients with PD were recorded simultaneously using 8 Optitrack prime 41/17W motion capture cameras sampled at 240 Hz and calibrated using Optitrack Motive 2.0.2 software (NaturalPoint, Corvallis, Oregon, USA) and two smartphone cameras (Galaxy A Quantum, Samsung) sampled at 30 Hz and positioned on the lateral side **(Fig 1A)** and near-frontal sides **(Fig 1B)** of the subject. The 39 markers were attached to the participants’ bodies by trained personnel (**Fig 1C**). The marker set used was the conventional whole-body marker set provided by Motive software [19]. Gait measurements included a 30-second treadmill walk and a 6-meter overground walk. For the overground walking measurements, subjects completed two round trips between the marked red lines shown in **Fig 1D**, during which motion capture and video data of their gait were collected. This resulted in the camera angle transitioning from front to rear (or rear to front) for the near-frontal view and from left to right (or right to left) for the lateral view as the subject changed walking direction. Both near-frontal and lateral views were taken in wide-angle mode to capture the entire 6-meter walking space. For the treadmill walking measurements, the subjects walked on a treadmill for 30 seconds at a self-determined comfortable speed. Prior to treadmill measurements, the subjects had the opportunity to practice and select the walking speed that felt most comfortable. Regarding gait speed, the subjects walked on a treadmill before the experiment and their usual gait speed was recorded.

### Data processing

The motion capture data were exported through Motive 2.0.2 software [20]. Smartphone videos recorded from the near-frontal and lateral sides were used to extract 2D joint locations using MediaPipe Pose. This is a top-down body pose estimation method that estimates 33 joint locations across the body from RGB frames and is a lightweight model for HPE optimized for real-time inference [21]. Post-processing steps included filtering and gap filling as needed to ensure data accuracy and reliability. For motion capture data, post-processing involved manually applying and correcting marker labels using Motive software to address incorrect labels, and performing cubic or pattern-based gap filling in cases where marker data was missing. Pose estimation data obtained from MediaPipe was denoised by creating gaps at points where the acceleration of each marker crossed a threshold, performing cubic interpolation, and applying a zero-lag 4^th^ order low-pass Butterworth filter with cut-off frequency at 5 Hz [22].

Heel-strike and toe-off points were initially calculated to derive the temporospatial gait parameters. Temporal gait parameters were calculated from the temporal information of the heel-strike and toe-off times using a previously described method based on peak detection of the ankle relative to the hip joint center coordinates [23]. Spatial gait parameters were calculated by measuring the pixel coordinates in the video and converting them into real-world distances using the known distance between two marked endpoints. Gait speed was calculated by dividing step length by step time. Spatial parameters were computed only from video data captured from the lateral view. To calculate the mean absolute error (MAE) and correlation coefficient for the joint angles, we downsampled the motion capture data to 30 frames per second (FPS) and synchronized it with the video by referencing the color variations observed in the motion capture camera during recording. The motion capture camera changes color from blue to green during filming, and the segments of the walking video corresponding to the green phase were trimmed. This process ensured that the duration of the motion capture data matched the length of the video recorded by the smartphone. The motion capture and smartphone camera sampling rates were 240 FPS and 30 FPS, respectively. Joint angles were calculated from the joint position data output by both the motion capture system and MediaPipe using MATLAB. Markers at the shoulder, hip, and knee, along with keypoints at the shoulder, hip, and knee, were used to calculate hip angles. Knee angles were calculated using markers and keypoints at the hip, knee, and ankle, while ankle angles were determined using markers and keypoints at the knee, ankle, and foot.

### Statistical analysis

Motion capture and HPE were used to calculate lower limb joint angles in the sagittal plane, temporal gait parameters (stride time, stance time, swing time, double support time, cadence), and spatial parameters (step length, walking speed). The error in our data was quantified by calculating the difference between gait parameters measured using HPE and the ground truth values obtained from motion capture. The MAE was computed for each participant by averaging the gait parameters across multiple cycles (Participants mean). Additionally, the analysis was performed using all gait cycles (All step). Both MAE and linear regression analysis were employed to assess the correlation (R) between the two methods, while the Bland-Altman plot was used to evaluate the agreement between HPE and motion capture measurements.

## Results

The clinical and demographic characteristics of the patients are shown in **Table 1**. The mean (±standard deviation) age and age at disease onset of the patients were 66.9±7.9 years and 64.1±8.2 years, respectively, and 12 (50%) patients were men. The mean duration of PD was 2.8±1.2 years, indicating the patients were in the early stages of the disease.

**Table 1:** Clinical and demographic characteristics of participants with PD.

Variables	Participants
Number of participants	24
Age, y	66.9 (7.9)
Male sex, %	12 (50.0%)
Age at PD onset, y	64.1 (8.2)
PD duration, y	2.8 (1.2)
MDS-UPDRS part 1 score	5.8 (4.0)
MDS-UPDRS part 2 score	4.4 (3.8)
MDS-UPDRS part 3 score	20.4 (6.6)
MoCA score	23.5 (3.2)
BDI score	7.4 (3.6)

Data are n (%) and the mean (standard deviation).

Abbreviations: BDI = Beck Depression Inventory; MDS-UPDRS = Movement Disorders Society Unified Parkinson’s Disease Rating Scale; MoCA = Montreal Cognitive Assessment.

**Fig 2:**
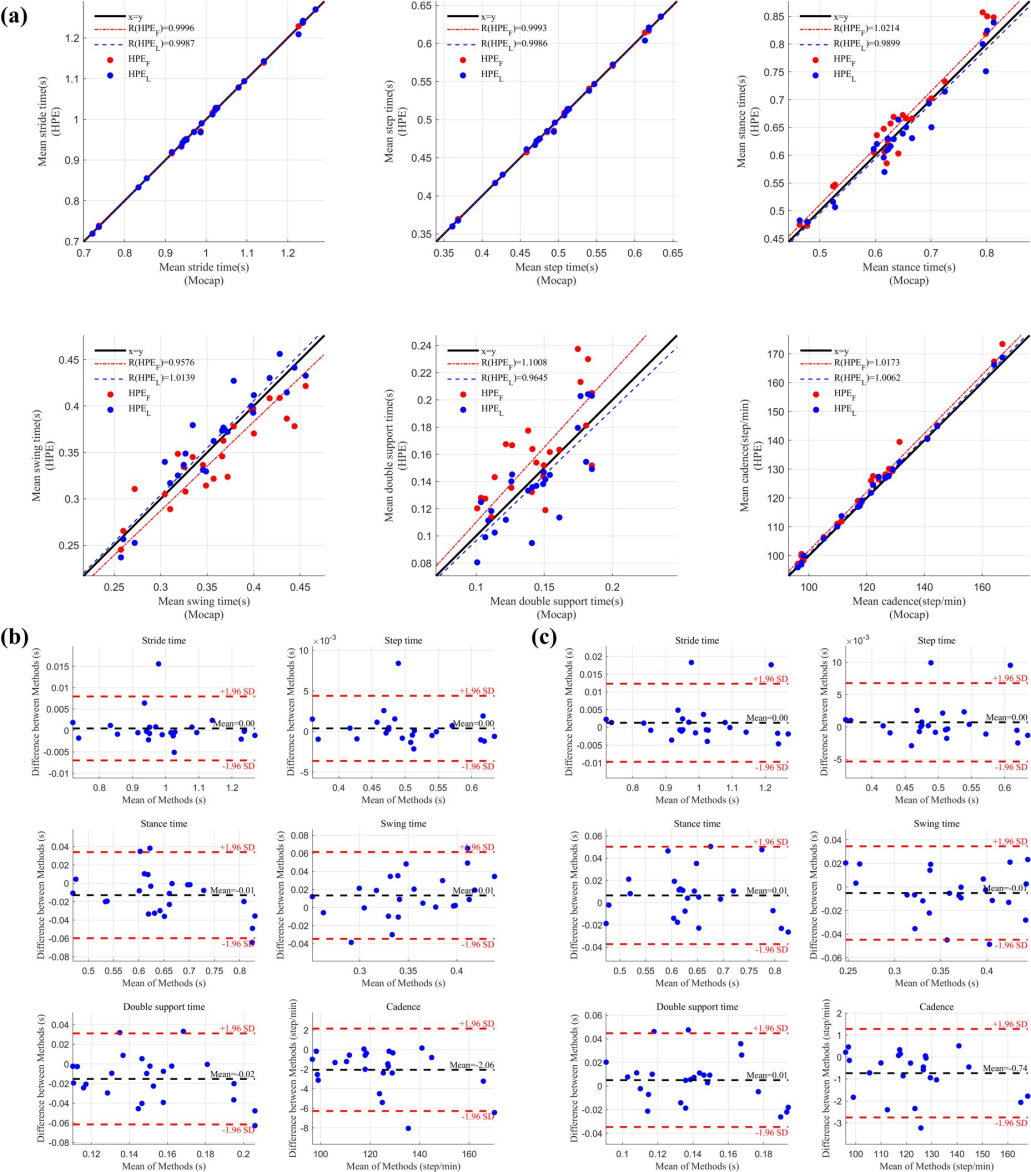
Comparison of temporal gait parameters between motion capture and pose estimation systems during treadmill walking. (a) Linear regression plots comparing temporal gait parameters obtained from motion capture (Mocap) with those from near-frontal and lateral video-based pose estimation (HPE_F_ and HPE_L_). Red dots represent comparisons between Mocap and near-frontal HPE_F_, while blue dots represent comparisons between Mocap and lateral HPE_L_. The black line indicates the identity line, and the red and blue lines show the linear regression lines for HPE_F_ and HPE_L_ respectively. (b) Bland-Altman plots comparing temporal gait parameters between Mocap and HPE_F_. (c) Bland-Altman plots comparing Mocap and HPE_L_. The black line in (b) and (c) represents the mean difference between the two methods, while the red dashed lines indicate the limits of agreement (±1.96 standard deviations). Blue dots in (b) and (c) represent the error distribution for the corresponding comparisons.

The temporospatial gait parameter values and MAE calculated from the treadmill gait measurements are displayed in **Table 2**, with calculations based on motion capture as the ground truth and the HPE as the prediction data. Comparing the near-frontal (HPE_F_) and lateral views (HPE_L_) for treadmill measurements **(Fig 2A)**, the average difference of stride and step times was almost no difference, while stance, swing, and double support times were 0.02 seconds. The error between motion capture and HPE_L_ in step length was 0.01 (m), and the difference in gait speed was 0.03 m/s. In each of the Bland-Altman plots (**Fig 2B and 2C**), the mean difference (black line) is close to zero, suggesting that there is no systematic bias between the two methods for most parameters. Most of the data lies within ±1.96 standard deviations (red line). However, there are two data points in the plot that are outside the standard deviation range.

**Table 2:** Comparison of video-based and motion capture measurements of spatiotemporal gait parameters from treadmill walking.

	Error (Mean±SD)		Linear regression		95% Limits of Agreements	
Variables	|Mocap-HPE_F_|	|Mocap-HPE_L_|	Mocap vs. HPE_F_	Mocap vs. HPE_L_	Mocap vs. HPE_F_	Mocap vs. HPE_L_
**Participants mean**						
**Stride Time (s)**	0.00±0.00	0.00±0.00	0.9996	0.9987	-0.007;0.008	-0.010;0.012
**Step Time (s)**	0.00±0.00	0.00±0.00	0.9993	0.9986	-0.004;0.004	-0.005;0.007
**Stance Time (s)**	0.02±0.02	0.02±0.01	1.0214	0.9899	-0.060;0.034	-0.037;0.050
**Swing Time (s)**	0.02±0.02	0.02±0.01	0.9576	1.0139	-0.035;0.061	-0.045;0.034
**Double Support Time (s)**	0.02±0.02	0.02±0.01	1.1008	0.9645	-0.062;0.031	-0.035;0.045
**Cadence (Step/min)**	2.08±2.13	0.88±0.90	1.0173	1.0062	-6.281;2.159	-2.751;1.280
**Step length (m)**	-	0.01±0.01	-	0.9576	-	-0.008;0.034
**Gait speed (m/s)**	-	0.03±0.02	-	0.9516	-	-0.022;0.076
**All step**						
**Stride Time (s)**	0.04±0.04	0.03±0.03	0.9999	0.9966	-0.108;0.107	-0.088;0.095
**Step Time (s)**	0.05±0.04	0.03±0.03	0.9991	0.9959	-0.129;0.129	-0.079;0.082
**Stance Time (s)**	0.05±0.05	0.04±0.04	1.0206	0.9575	-0.145;0.117	-0.073;0.127
**Swing Time (s)**	0.04±0.04	0.04±0.03	0.9594	1.0633	-0.099;0.125	-0.106;0.059
**Double Support Time (s)**	0.06±0.05	0.04±0.03	1.0826	0.8299	-0.154;0.124	-0.055;0.103
**Cadence (Step/min)**	14.07±15.24	8.07±12.19	1.0147	1.0091	-42.520;38.406	-29.750;27.372
**Step length (m)**	-	0.03±0.02	-	0.952	-	-0.059;0.083
**Gait speed (m/s)**	-	0.07±0.05	-	0.9434	-	-0.135;0.185

Mocap, motion capture; HPE_L_, lateral view camera; HPE_F_, near-frontal view camera.

MAE are the mean±standard deviation.

The comparison between participants mean and all step revealed that participants mean consistently yielded smaller errors across all parameters. For example, the step time MAE was lower in the participants mean analysis compared to all step, indicating that averaging across all participants reduces variability. This indicates that the pose estimation system may be suitable for capturing averaged gait parameters across multiple trials; however, certain parameters exhibit limits of agreement in the range of approximately -20% to +20%, highlighting limitations in precision for stride-by-stride analysis.

The linear regression analysis showed high correlation coefficients, particularly for temporal parameters, with Bland-Altman plots confirming minimal bias. Spatial parameters such as step length and gait speed exhibited slightly wider limits of agreement, but the differences were within clinically acceptable ranges, indicating that HPE_L_ provides a reliable alternative for treadmill gait analysis, especially for temporal parameters.

**Fig 3:**
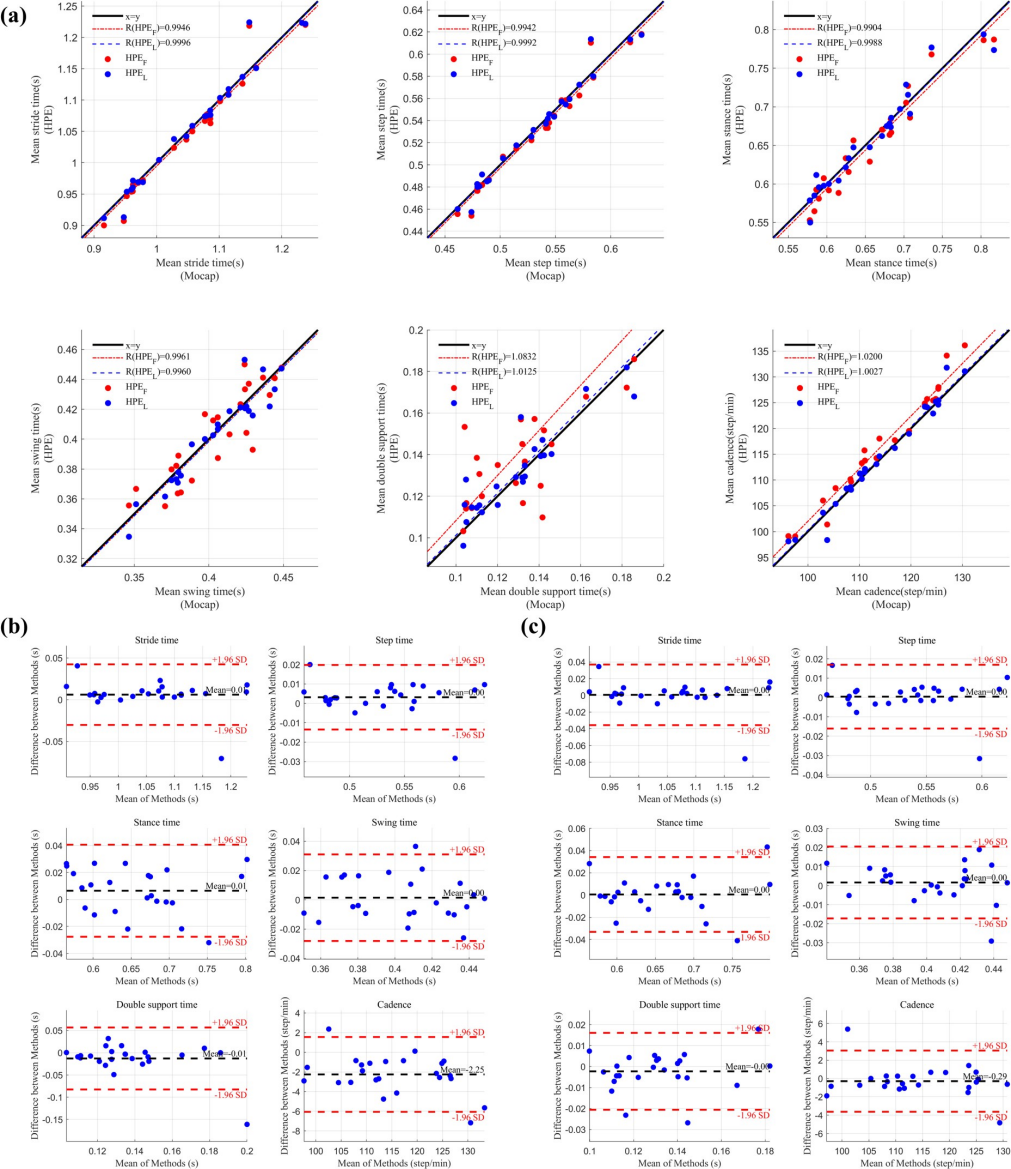
Comparison of temporal gait parameters between motion capture and pose estimation systems during backward overground walking. (a) Linear regression plots comparing temporal gait parameters obtained from motion capture (Mocap) with those from near-frontal and lateral video-based pose estimation (HPE_F_ and HPE_L_). Red dots represent comparisons between Mocap and near-frontal HPE_F_, while blue dots represent comparisons between Mocap and lateral HPE_L_. The black line indicates the identity line, and the red and blue lines show the linear regression lines for HPE_F_ and HPE_L_ respectively. (b) Bland-Altman plots comparing temporal gait parameters between Mocap and HPE_F_. (c) Bland-Altman plots comparing Mocap and HPE_L_. The black line in (b) and (c) represents the mean difference between the two methods, while the red dashed lines indicate the limits of agreement (±1.96 standard deviations). Blue dots in (b) and (c) represent the error distribution for the corresponding comparisons.

**Fig 4:**
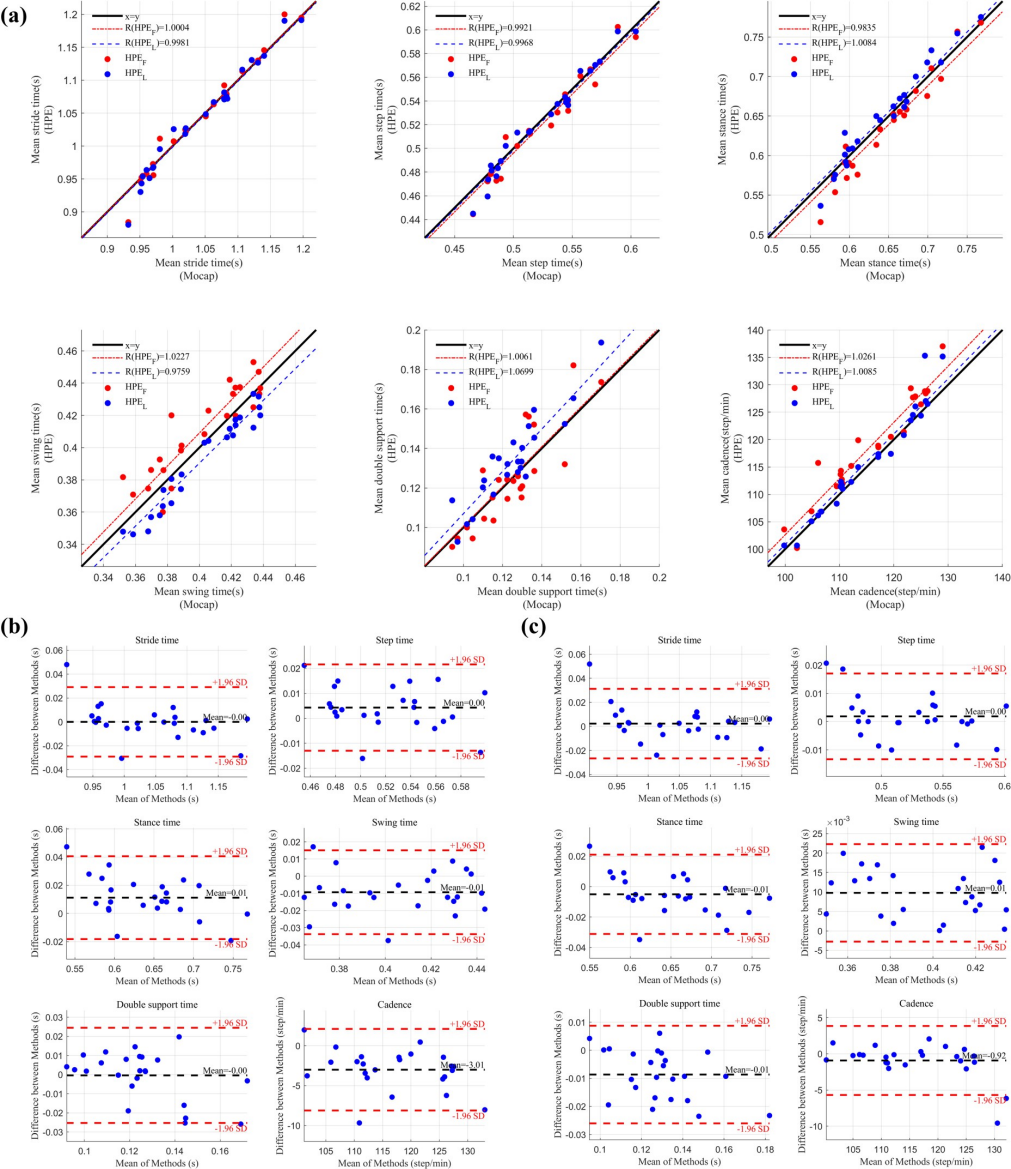
Comparison of temporal gait parameters between motion capture and pose estimation systems during forward overground walking. (a) Linear regression plots comparing temporal gait parameters obtained from motion capture (Mocap) with those from near-frontal and lateral video-based pose estimation (HPE_F_ and HPE_L_). Red dots represent comparisons between Mocap and near-frontal HPE_F_, while blue dots represent comparisons between Mocap and lateral HPE_L_. The black line indicates the identity line, and the red and blue lines show the linear regression lines for HPE_F_ and HPE_L_ respectively. (b) Bland-Altman plots comparing temporal gait parameters between Mocap and HPE_F_. (c) Bland-Altman plots comparing Mocap and HPE_L_. The black line in (b) and (c) represents the mean difference between the two methods, while the red dashed lines indicate the limits of agreement (±1.96 standard deviations). Blue dots in (b) and (c) represent the error distribution for the corresponding comparisons.

The comparison of temporospatial gait parameters during overground walking between video-based HPE and motion capture demonstrated minimal errors (**Table 3** and **Figs 3 and 4**). For step time, the MAE was 0.01 ± 0.01 seconds for both HPE_F_ and HPE_L_ views during forward walking, and 0.01 ± 0.01 seconds during backward walking. For step length, measured from the HPE_L_, the error was 0.09 ± 0.05 meters for forward walking and 0.07 ± 0.03 meters for backward walking.

**Table 3:** Comparison of video-based and motion capture measurements of spatiotemporal gait parameters from overground walking.

		Mean absolute error (MAE)		Linear regression (R)		95% Limits of Agreements (LoA)	
Variables	Gait direction	|Mocap-HPE_F_|	|Mocap-HPE_L_|	Mocap vs. HPE_F_	Mocap vs. HPE_L_	Mocap vs. HPE_F_	Mocap vs. HPE_L_
**Participants mean**							
**Stride Time (s)**	Forward	0.01±0.01	0.01±0.01	1.0004	0.9981	-0.029;0.029	-0.027;0.031
	Backward	0.01±0.02	0.01±0.02	0.9946	0.9996	-0.030;0.042	-0.036;0.037
**Step Time (s)**	Forward	0.01±0.01	0.01±0.01	0.9921	0.9968	-0.013;0.022	-0.013;0.017
	Backward	0.01±0.01	0.01±0.01	0.9942	0.9992	-0.013;0.020	-0.016;0.017
**Stance Time (s)**	Forward	0.01±0.01	0.01±0.01	0.9835	1.0084	-0.018;0.041	-0.031;0.021
	Backward	0.02±0.01	0.01±0.01	0.9904	0.9988	-0.028;0.041	-0.033;0.034
**Swing Time (s)**	Forward	0.01±0.01	0.01±0.01	1.0227	0.9759	-0.034;0.015	-0.003;0.022
	Backward	0.01±0.01	0.01±0.01	0.9961	0.996	-0.028;0.031	-0.017;0.021
**Double Support Time (s)**	Forward	0.01±0.01	0.01±0.01	1.0061	1.0699	-0.025;0.024	-0.026;0.009
	Backward	0.02±0.03	0.01±0.01	1.0832	1.0125	-0.083;0.057	-0.021;0.016
**Cadence (Step/min)**	Forward	3.21±2.34	1.48±2.13	1.0261	1.0085	-8.113;2.095	-5.692;3.852
	Backward	2.46±1.66	1.09±1.32	1.02	1.0027	-6.062;1.556	-3.633;3.048
**Step length (m)**	Forward	-	0.09±0.05	-	1.129	-	-0.219;0.066
	Backward	-	0.07±0.03	-	1.0907	-	-0.153;0.047
**Gait speed (m/s)**	Forward	-	0.17±0.09	-	1.1226	-	-0.393;0.111
	Backward	-	0.13±0.06	-	1.0922	-	-0.296;0.092
**All step**							
**Stride Time (s)**	Forward	0.02±0.02	0.02±0.03	1.001	1.0016	-0.064;0.063	-0.076;0.072
	Backward	0.02±0.02	0.03±0.04	1.0014	1.0069	-0.063;0.061	-0.094;0.079
**Step Time (s)**	Forward	0.05±0.03	0.02±0.02	0.9894	0.9948	-0.114;0.126	-0.052;0.057
	Backward	0.05±0.03	0.02±0.02	0.9956	1.0008	-0.114;0.119	-0.054;0.053
**Stance Time (s)**	Forward	0.03±0.03	0.02±0.02	0.9849	1.0111	-0.070;0.089	-0.067;0.051
	Backward	0.03±0.02	0.02±0.03	0.999	1.0066	-0.076;0.078	-0.069;0.060
**Swing Time (s)**	Forward	0.03±0.03	0.02±0.02	0.9979	0.9734	-0.085;0.086	-0.053;0.072
	Backward	0.03±0.03	0.02±0.02	0.9854	0.9877	-0.068;0.080	-0.047;0.056
**Double Support Time (s)**	Forward	0.05±0.03	0.02±0.01	0.9371	1.001	-0.105;0.120	-0.045;0.045
	Backward	0.06±0.03	0.02±0.01	1.017	0.9772	-0.140;0.134	-0.043;0.049
**Cadence (Step/min)**	Forward	11.19±6.68	4.47±4.87	0.9961	0.9875	-24.956;26.145	-11.382;14.033
	Backward	10.75±6.14	4.66±5.28	0.9997	0.9851	-24.278;24.316	-11.961;15.004
**Step length (m)**	Forward	-	0.11±0.07	-	0.8367	-	-0.094;0.268
	Backward	-	0.10±0.06	-	0.9245	-	-0.176;0.255
**Gait speed (m/s)**	Forward	-	0.21±0.14	-	0.8439	-	-0.204;0.533
	Backward	-	0.19±0.12	-	0.9271	-	-0.332;0.482

Mocap, motion capture; HPE_L_, lateral view camera; HPE_F_, near-frontal view camera.

MAE are the mean±standard deviation.

A comparison of participants mean and all step values revealed differences. Participants mean reflects the average step time and step length across all participants, while all step includes the error calculated across all individual steps. The step time MAE was slightly higher for all steps (0.02 ± 0.02 seconds) compared to participants mean (0.01 ± 0.01 seconds), indicating more variability when considering each gait cycle individually.

The Bland-Altman plots showed that most data points fell within ±1.96 standard deviations of the mean difference, suggesting no significant systematic bias between HPE and motion capture methods.

The MAE and correlation coefficient of the measured hip, knee, and ankle angles were derived from the analysis results of the images taken from the side (Table 4). Forward overground walking was taken from the participant’s right side, and backward overground walking and treadmill walking were taken from the participant’s left side. The analysis results showed that the joints closer to the camera showed higher accuracy. Specifically, when taken from the left side, the MAE of the left joint was lower and the correlation coefficient was higher, which means that the tracking and analysis accuracy of the closer joint was better.

**Table 4:** Range of motions mean absolute error, and correlation coefficients of Joint angle.

			Left hip	Right hip	Left knee	Right knee	Left ankle	Right ankle
Range of motions	Overground	Forward	29.2±4.1	29.7±3.9	55.6±4.3	54.3±4.8	24.1±4.8	25.5±4.3
		Backward	28.7±4.1	29.1±3.6	55.2±4.3	54.3±5.3	24.1±4.4	25.5±4.7
	Treadmill		20.0±5.7	21.0±5.4	41.1±9.1	40.5±8.1	12.3±4.2	14.3±4.6
Mean absolute error	Overground	Forward	1.41±0.28	1.74±0.44	3.11±0.64	3.22±0.55	3.35±0.80	3.23±0.60
		Backward	1.80±0.64	1.57±0.50	3.28±0.67	3.64±0.65	3.01±0.78	2.43±0.90
	Treadmill		1.69±0.68	2.76±1.80	3.36±0.84	4.31±1.64	1.81±0.64	3.47±1.05
Correlation coefficients	Overground	Forward	0.99±0.00	0.98±0.01	0.98±0.01	0.98±0.01	0.79±0.07	0.81±0.07
		Backward	0.98±0.01	0.99±0.01	0.97±0.01	0.98±0.01	0.81±0.09	0.90±0.07
	Treadmill		0.96±0.05	0.82±0.34	0.97±0.02	0.94±0.10	0.67±0.35	0.27±0.43

**Fig 5:**
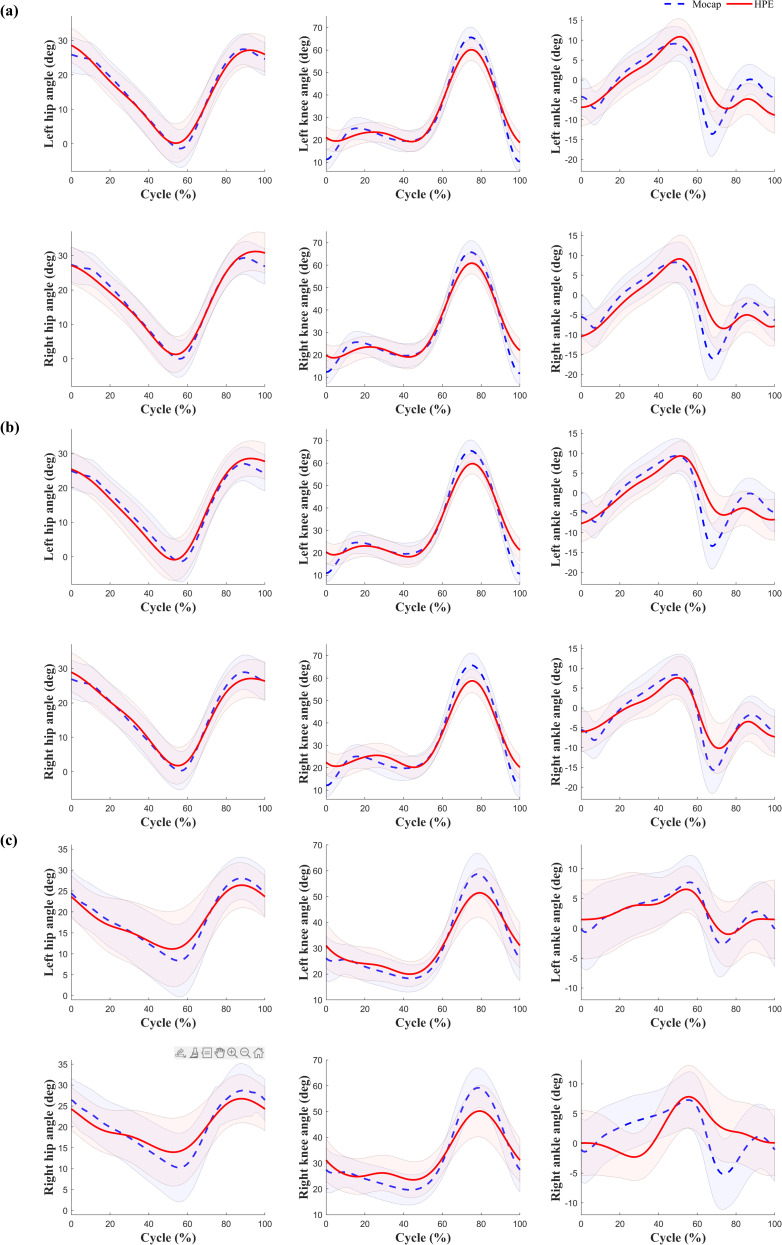
Sagittal plane joint angle analysis of the hip, knee, and ankle during overground and treadmill walking. The graphs compare joint angle trajectories for different conditions: (a) Forward walking overground, (b) Backward walking overground, (c) Treadmill walking. The solid red lines represent the mean joint angles of HPE_L_. The blue dashed line represents joint angle from Mocap. The shaded areas indicate the standard deviation across gait cycles.

**Fig 5** and **Table** 4 compare the MAE and correlation coefficients for forward and backward walking. There was no significant difference in the size of the MAE and correlation coefficient according to the walking direction (forward vs. backward), but a slight asymmetry was observed depending on the lateral shooting position. In backward overground walking and treadmill walking, the MAE of the left joint was lower, and the correlation coefficient was higher when taken from the participant’s left side, which suggests that the measurement accuracy of this joint was higher. Conversely, during Forward Overground Walking, the MAE of the right joint was found to be lower and the correlation coefficient higher when the images were taken from the right side of the participant. These results demonstrate that the camera position has a significant effect on the joint angle measurements in images taken from the side.

## Discussion

In this study, gait analysis of patients with PD was conducted using 3D motion capture and a smartphone camera with a pose estimation algorithm positioned at the near-frontal and lateral sides of the subjects. Gait analysis involved treadmill and overground measurements, and joint position data were extracted from videos captured with a smartphone using the HPE algorithm. Temporospatial gait parameters and joint angles in the sagittal plane were calculated from the HPE data. We computed the MAE, correlation coefficients, and 95% Limits of Agreement between the motion capture and HPE-calculated temporospatial gait parameters and kinematic characteristics in the sagittal plane.

Motion capture presents certain limitations, such as the requirement for expensive equipment and dedicated space for measurements, making motion capture less accessible [24, 25]. In contrast, pose estimation enables gait analysis using readily available devices like webcams or smartphones, without the need for specific setup space [26]. MediaPipe Pose [27], a lightweight model compared to OpenPose [22], operates seamlessly across devices and supports real-time processing in both mobile and desktop environments. This makes it optimal for single-person pose analysis, as used in this study.

Cameras were placed on the subject’s lateral and near-frontal sides to measure gait both on a treadmill and overground in people with PD. A motion capture system was also used to assess the accuracy of the pose estimation. Both near-frontal and lateral view recordings showed errors of less than 0.033 seconds, which corresponds to less than the time for one frame at the current frame rate. To achieve even greater accuracy, recording at a higher frame rate would be necessary. Accurate measurement of gait parameters is crucial in various applications, including biomechanical studies and clinical gait analysis [28].

To quantify the spatial parameters, it is important to measure the forward displacement of the foot in the anterior-posterior direction. It was not possible to calculate these parameters using HPE data from the near-frontal view obtained in this study. In the lateral view, the step length showed discrepancies of approximately 0.10 m during overground walking and about 0.03 m during treadmill walking. The larger error observed in overground measurements can be attributed to increased distortion caused by the use of a wide-angle camera, which is necessary to capture the entire walking distance. Considering the minimal clinically important difference for step length in PD patients on a treadmill without a wide-angle camera, the step length error observed in this study is within the acceptable range for clinical relevance, at 0.03±0.02 (m) [29]. This suggests that, once camera distortion is addressed, differences in stride length measured by HPE_L_ do not significantly affect clinical interpretation of gait parameters in this population.

Videos captured from the lateral view were used to estimate the joint angles. Specifically, the left joint angles exhibited higher accuracy than the corresponding right joint angles in the lateral view setting for treadmill measurements. For treadmill gait measurement, a lateral-view video was captured from the subject’s left side. The right joint was obscured owing to body occlusion, resulting in low visibility (**Table** 5). The accuracy of the left joint, which had a relatively high detection rate influenced by visibility, surpassed that of the right joint. measurements during overground walking showed better accuracy compared to treadmill walking. This can be attributed to less body occlusion during overground walking, which allowed for better joint visibility compared to the treadmill setup. The MAE and correlation coefficient for the ankle joint were notably worse compared to those for the hip and knee joints, indicating greater difficulty in capturing accurate ankle movements due to factors such as occlusion and camera placement angle. These joint-specific differences in tracking accuracy are clinically significant, as accurate joint tracking is essential for evaluating specific gait abnormalities, especially in conditions like PD where subtle impairments in joint movement may be crucial for diagnosis and progression assessment. Using multiple cameras allows for gait analysis from various perspectives. However, when analyzing gait kinematics, analyzing overground walking proves to be more effective than treadmill walking, as it reduces issues related to body occlusion and improves joint visibility, which is critical for capturing subtle joint movements in conditions like PD.

**Table 5:** Visibility of each joint in treadmill and overground measurement videos.

Experiment method	Visibility location	Near-frontal		Lateral	
		Left	Right	Left	Right
Treadmill	Shoulder	1.0000	1.0000	1.0000	**0.9998**
	Hip	1.0000	1.0000	1.0000	1.0000
	Knee	0.9470	0.9650	0.9183	0.2738
	Ankle	0.9472	0.9652	0.9393	0.5758
	Heel	0.7816	0.7316	0.8223	0.6310
	Toe	0.9594	0.9582	0.9413	0.7237
Over-ground (Forward direction)	Shoulder	0.9999	0.9995	0.9995	0.9999
	Hip	0.9997	0.9993	0.9983	0.9994
	Knee	0.9878	0.8507	0.8689	0.9842
	Ankle	0.9859	0.9053	0.8912	0.9797
	Heel	0.9821	0.9113	0.9033	0.9823
	Toe	0.9430	0.8094	0.7563	0.9267
Over-ground (Backward direction)	Shoulder	0.9998	1.0000	0.9999	0.9995
	Hip	0.9994	0.9996	0.9994	0.9983
	Knee	0.9180	0.9693	0.9844	0.8693
	Ankle	0.9133	0.9575	0.9800	0.8931
	Heel	0.8620	0.7999	0.9826	0.9048
	Toe	0.9296	0.9513	0.9278	0.7616

This study used video and motion capture walking data of patients with PD. Thus, the accuracy of gait analysis using pose estimation was evaluated. Patients with PD show differences in gait characteristics compared to healthy people, such as reduced walking speed, sometimes dragging feet on the ground, and trembling feet [30, 31]. The validity of gait analysis in patients with PD was confirmed through pose estimation. In future research, video data from walking patients with PD will be used instead of visiting a motion-capture-equipped laboratory for gait measurements. This approach eliminates the need for patient transportation, enhances study efficiency, and provides greater convenience, enabling a larger subject pool to be evaluated. The lateral view is advantageous for gait analysis as it allows for the analysis of both temporospatial parameters and kinematics, making it more effective than the near-frontal view. However, capturing lateral view recordings requires 2 to 3 meters of space to the side of the walking subject, which may not be feasible in confined spaces like narrow hallways. In such scenarios, recording from the near-frontal view may be more practical and advantageous for gait analysis.

This study demonstrates the potential of HPE in assessing gait parameters in patients with PD, but several challenges remain to be addressed before this technology can be fully integrated into clinical practice. For instance, the observed reduction in gait speed and stride length in PD patients aligns with existing literature that compares PD gait characteristics to those of healthy controls, emphasizing the importance of targeting these parameters in rehabilitation programs. These findings suggest that while the current pose estimation system is effective for capturing average gait parameters across multiple trials, it may not yet be precise enough for detailed, real-time stride-by-stride assessment. Key areas of future research include addressing bias in the training dataset [32], which can lead to inaccuracies or limited generalizability, particularly across diverse patient populations. Additionally, there is a need to explore the differences between supervised learning approaches commonly used in controlled settings and consumer-oriented ‘plug-and-play’ architectures that offer broader accessibility but may compromise accuracy and reliability. Camera calibration techniques are expected to enhance the accuracy of spatial parameters by addressing distortion caused by wide-angle cameras [33]. Future studies should aim to recruit larger and more diverse cohorts to create a more generalized dataset, enhancing the robustness and applicability of HPE in clinical settings. Furthermore, applying HPE to other neurological conditions, such as stroke or multiple sclerosis, could broaden its clinical utility and help validate its effectiveness across different patient populations.

## Conclusion

This study demonstrated that pose estimation can be used with readily available video recording devices to reasonably estimate spatiotemporal measurements and joint angles from PD patients walking overground and on a treadmill. When collecting video gait data, it is crucial to select an appropriate camera position and angle. While both near-frontal and lateral views have advantages, research has demonstrated that near-frontal view recordings with clear visibility of the heel and toe markers are more accurate for temporal gait parameter estimation. By contrast, lateral view recordings are considered superior in terms of spatial gait parameters and joint angle estimation. In confined clinical settings where recording from a lateral view may not be feasible, recordings from a near-frontal view can serve as a viable alternative.
